# Prevalence and Related Factors of Euthyroid Sick Syndrome in Children with Untreated Cancer According to Two Different Criteria

**DOI:** 10.4274/jcrpe.0015

**Published:** 2018-07-31

**Authors:** Ali Duyu, Elvan Çağlar Çıtak, Erdem Ak, Serhan Küpeli, Begül Yağcı Küpeli, İbrahim Bayram, Gülay Sezgin, Gülçin Eskendari, Kerem Sezer

**Affiliations:** 1 Mersin University Faculty of Medicine, Department of Pediatric Oncology, Mersin, Turkey; 2 Çukurova University Faculty of Medicine, Department of Pediatric Oncology, Adana, Turkey; 3 Adana City Training and Research Hospital, Clinic of Pediatric Oncology, Adana, Turkey; 4 Mersin University Faculty of Medicine, Department of Medical Biochemistry, Mersin, Turkey; 5 Mersin University Faculty of Medicine, Department of Internal Medicine, Division of Endocrinology and Metabolism, Mersin, Turkey

**Keywords:** Euthyroid sick syndrome, children, cancer, interleukin 6, interleukin 8, tumor necrosis factor alpha

## Abstract

**Objective:**

In this study, we evaluated the frequency of euthyroid sick syndrome (ESS) among patients with childhood cancer and its association with the stage of disease, nutritional parameters and cytokines levels.

**Methods:**

Eighty newly diagnosed children were included in the study. ESS was assessed in two different ways. According to criteria 1 ESS was present if free triiodothyronine (fT3) was below the lower limit and free thyroxine was within the normal or low limits, thyroid-stimulating hormone (TSH) was in the normal range. According to criteria 2, in addition to the above, it was required that reverse triiodothyronine (rT3) be performed and was higher than normal limits.

**Results:**

Three of our pediatric patients had subclinical hypothyroidism and two had subclinical hyperthyroidism. Out of 75 patients, ESS was identified in 14 (17.3%) according to criteria 1 and in eight (10.6%) according to criteria 2. Only fT3 levels were significantly different in the ESS (+) and ESS (-) groups (p<0.05) according to criteria 1. A significantly negative correlation between interleukin (IL)-6 and fT3 was found, according to both sets of criteria. tumor necrosis factor alpha was negatively correlated with fT3 levels only in the criteria 1 group. There were no correlations between IL-1β and fT3, free thyroxine, rT3 and TSH levels.

**Conclusion:**

ESS may occur in childhood cancer and thyroid function testing should be performed routinely when cancer is diagnosed.

## What is already known on this topic?

Euthyroid sick syndrome is seen in patients with cancer. Euthyroid sick syndrome has been associated with a worse prognosis in cancer patients. 

## What this study adds?

Up to now, it has not been recognized that children with cancer might have contemporaneous euthyroid sick syndrome at the time of cancer diagnosis. To our knowledge, this is the first study to investigate euthyroid sick syndrome prevalence at the time of diagnosis of a number of different types of pediatric cancers. The prevalence of euthyroid sick syndrome in a range of different cancer types ranged from 11 to 17% depending on the definition of euthyroid sick syndrome used.

## Introduction

Euthyroid sick syndrome (ESS), also known as non-thyroidal illness syndrome or low triiodothyronine (T3) syndrome, is characterized by alterations in the levels of thyroid hormones due to non-thyroidal diseases in the absence of any disorder related to the hypothalamic-hypophysial axis or thyroid gland (1,2). An imbalance between the activities of types I and II deiodinase, decreased sensitivity of the hypothalamus and pituitary gland to thyroid hormones and reduced T4 protein binding and cellular uptake have been proposed for the pathogenesis of the syndrome, which is not well understood as yet (3,4). Oxidative stress and increased cytokines such as interleukin (IL)-6 and tumor necrosis factor-alpha (TNF-α), are among the factors possibly contributing to the development of the syndrome (5,6).

It has been much debated whether ESS represents a physiological adaptive response to systemic illness or conversely a maladaptive state at the tissue level (3). ESS has been described in liver disease, renal failure, after stress or surgery, in the sick elderly, in malnutrition and in malignancies (7). It is also reported that the presence of ESS is not associated with the type of the underlying disease but instead on its severity (7,8). 

There is scant knowledge about cancer and ESS in adult patients (9,10,11,12,13). Mohn et al (14) have investigated ESS prevalence in seven children with Hodgkin disease. However, no research to date has focused on the incidence of ESS in childhood cancer. 

In the present study, we aimed to determine the frequency of ESS, to identify its relation with hematological parameters, with body mass index (BMI) and with serum albumin levels. A further aim was to investigate its association with the stage of the disease and the relationship between cytokine levels, IL-6, TNF-α and IL-1β in childhood cancer patients.

## Methods

Eighty consecutive patients with histologically diagnosed childhood cancer from three pediatric oncology centers presenting between January 2015 and December 2016 were enrolled in this study. Exclusion criteria were the following: intrinsic thyroid or pituitary-hypothalamic disease, use of special drugs known to affect serum thyroid hormone concentration such as glucocorticoids, amiodorone, β blockers, sucralfate, phenytoin, salicylates and rifampin, and presence of diseases such as secondary malignancy, diabetes mellitus, nephrotic syndrome, chronic hepatic or renal disease and other systemic infectious diseases associated with thyroid function anomalies. The subjects underwent thyroid function tests, nutritional evaluation and staging of the disease.

This study was approved by the Ethics Committee of Mersin University (grant no: 290-2015). Written informed consent was obtained from each patient/patient’s family.

Blood samples were obtained between 08.00 and 10.00 am after overnight fasting and the serum samples were stored, frozen at -70 °C, until analysis. Free T3 (fT3), free thyroxine (fT4) and thyroid stimulating hormone (TSH) parameters were measured by electro chemiluminescence immunoassay kits (Modular Cobas 6000, Roche Diagnostics, GmbH, Mannheim, Germany). Serum IL-1β, IL-6, TNF-α and reverse T3 (rT3) was assayed by enzyme-linked immunosorbent assay (ELISA) (DSX Automated ELISA, Dynex Technologies, GmbH, Denkendorf, Germany). Reference ranges are 1.71-3.7 pg/mL for fT3, 0.7-1.48 ng/dL for fT4, and 0.34-5.6 mIU/mL for basal serum TSH. Normative value for rT3 was obtained from the manufacturers as being in the range 2.4-33.6 ng/dL. Patients with normal fT3, fT4 and TSH were considered euthyroid and those who did not have normal fT3, fT4 and TSH were diagnosed as cases of thyroid dysfunction. Thyroid function abnormalities were categorized according to serum TSH and free thyroid hormone levels as follows; i) euthyroidism: normal fT3, fT4 and TSH levels, ii): hypothyroidism: TSH elevation with decreased fT3 and fT4 levels, iii) hyperthyroidism: elevated fT3 and fT4 with suppressed TSH, iv): subclinical hypothyroidism: TSH elevation with normal fT3 and fT4, v): subclinical hyperthyroidism: low TSH with normal fT3 and fT4. Age specific reference values were used to assess fT3, fT4 and TSH levels (15). Thyroid hormone levels were reassessed in patients diagnosed with subclinical hypothyroidism in order to confirm the diagnosis and similar results were obtained.

ESS can be defined in two different ways. In order to be able to determine which of these two different definitions are more effective, we used two different ESS definitions; i): decreased fT3, normal or decreased fT4 and normal TSH [fT3 based definition (criteria 1)] (10,11), ii): decreased fT3, normal or decreased fT4, normal TSH and elevated rT3 [rT3 based definition (criteria 2)] (16).

### Nutritional and Biochemical Evaluation

Anthropometric and biochemical measurements were performed to assess the nutritional and biochemical state of the cases. For anthropometric measurement, weight and height were measured and BMI [weight (kg)/height (m^2^)] was calculated. Weight, height and BMI data were expressed as standard deviation scores (SDS) and compared with age- and sex-matched charts for Turkish children (17). Biochemical measurements included albumin and hemoglobin concentrations and leukocyte and platelet count. Serum albumin levels were measured using Abbot-labeled kits (catalog no: 30-3050/R2) in a Beckman Coulter-Synchron LX-20 chemistry auto analyzer device in the biochemistry laboratory of our hospital.

### Tumor Staging

Cases with cancer were staged according to their diagnosis. Hodgkin disease was staged according to the Ann–Arbor classification system (18). For non-Hodgkin lymphoma, the Murphy classification was used (19). International Neuroblastoma Staging System was used for patients with neuroblastoma (20). Patients with hepatoblastoma and hepatocellular cancer were staged using the PRETEXT system of the International Society of Pediatric Oncology (SIOP) (21). Wilms tumor was staged using the SIOP 2001 clinical staging system (22). Ewing sarcoma and osteosarcoma were staged according to the Enneking system (23). Patients with retinoblatoma were staged according to the international retinoblastoma staging system (24). Patients with nasopharyngeal carcinoma were staged by using the Tumor Node Metastasis system proposed by the “American Joint Committee on Cancer” (25).

### Treatment Response

The response criteria were defined as follows:

**Complete response:** Disappearance of all clinical and radiological evidence of disease.

**Very good partial response:** Primary mass reduced by more than 90%, no evidence of distant disease.

**Partial response:** Reduction of at least 50% of tumor size without evidence of new lesions.

**Stable disease:** Decrease of tumor size less than 50% or increase of tumor size less than 25%.

**Progressive disease:** More than 25% increase in any tumor size and/or appearance of new lesions.

### Statistical Analysis

The data were analyzed using the MedCalc Packet program. Normality of the data was checked by using the Shapiro-Wilk test. The data were evaluated using descriptive statistical methods (mean ± standard deviation, median, frequencies, and percentages). For intergroup comparison of categorical variables, a chi-square test was used. Comparisons between groups were done by Mann-Whitney U test or independent sample t test where appropriate. Spearman’s and Pearson correlation coefficient was used to assess relationships between thyroid hormone levels and IL-1, IL-6 and TNF-α. Significant differences (two-tailed p values) of <0.05 were regarded as significant.

## Results

A total of 80 children (47 boys, 33 girls) with a mean age of 7.07±5.04 (range: 2 months-16 years) were included in the study. The patients had different types of childhood cancers of different stages. Patient characteristics and laboratory findings of the study group are shown in Table 1. No patient had an elevation in thyroid autoantibody levels (anti-thyroid peroxidase and anti-thyroglobulin antibodies) and all patients had normal thyroid ultrasonography findings. In this series, three children were diagnosed as subclinical hypothyroidism and two as subclinical hyperthyroidisms. Thyroid hormone levels of children with subclinical hypothyroidism and subclinical hyperthyroidism are presented in Table 2. The remaining 75 children were clinically euthyroid. In the total group, fT3 (pg/mL), fT4 (ng/dL), TSH (mIU/mL) and rT3 (ng/dL) levels were 2.81±1.10, 1.24±0.35, 3.13±1.83 and 32.72±11.97, respectively. Out of 75 patients, ESS was identified in 14 (17.3%) according to criteria 1 and in eight (10.6%) according to criteria 2. It was found that there was no statistically difference between fT4 and TSH levels according to criteria 1 (p>0.05) but fT3 level was a statistically lower in the ESS (+) group according to criteria 1 (p<0.05) (Table 3). Also, there were no statistically significant differences in serum albumin and hemoglobin concentrations and in the white blood cell and platelet counts of patients with or without ESS in either group (Table 4). At the same time, there was no statistical difference in patients with or without ESS in terms of sex, stage of disease, weight, weight SDS, height, height SDS, BMI, and BMI SDS when comparing according to criteria (Table 4).

Thyroid hormone levels of nine out of 14 patients who were diagnosed as ESS by criteria 1 reverted to normal values after induction therapy. In five patients, recurrent/refractory disease was detected at follow-up. One of these patients was refractory Hodgkin’s lymphoma and was evaluated as progressive disease after two cycles of treatment. The others were relapsed neuroblastoma, Ewing sarcoma, osteosarcoma and rhabdomyosarcoma, respectively. These patients were found to respond partially to treatment when assessed after induction therapy. Patients without ESS were found to have complete or very good partial response after induction therapy according to criteria 1. The only relapsed case was one patient without ESS, and this patient was followed up with a diagnosis of neuroblastoma. Seven of eight ESS patients identified according to criteria 2 had normal thyroid hormone levels after induction therapy but only one patient with neuroblastoma was diagnosed as ESS according to criteria 2 and did not have a normal level of thyroid hormone after induction therapy and had recurrent disease at follow-up. IL-6, IL-1β and TNF-α levels for each criteria are shown in Table 5. We observed a significant negative correlation, using Pearson’ correlation coefficient between IL-6 and fT3 (r=-0.733, p=0.003) in patients with ESS according to criteria 1. There was a similar correlation between IL-6 and fT3 (r=-0.836, p=0.01) in patients with ESS according to criteria 2. Likewise, TNF-α was negatively and significantly correlated with lowered fT3 levels according to criteria 1 (r=-0.744, p=0.002) but no significant correlation was found according to criteria 2 (r=-0.195 and p=0.64). Also, there were no correlations between IL-1β and fT3, fT4, rT3 and TSH levels.

## Discussion

ESS can be described as abnormal thyroid function test results that occur in the setting of a non-thyroidal illness and in the absence of pre-existing hypothalamo-pituitary and thyroid gland dysfunction. ESS may be regarded as an acute phase response of the organism that serves as one of the major mechanisms to restore homeostasis in severe illness. The most typical alterations are low T3, low or normal T4, or elevated rT3 in the presence of normal TSH levels. However, it has been noted that fT3-based definitions are used for ESS diagnosis rather than rT3-based definitions (16). While evaluating an entity about which there is no consensus on identification and even given name (ESS or non-thyroidal illness), we decided to assess two different diagnostic criteria that were present in our study. In the studies, the presence of ESS was reported as an adverse factor to prognosis in different diseases. There is no consensus on whether this disease should be treated. For this reason, we wanted to determine the differences between the two diagnostic criteria in the diagnosis, follow-up, and prognosis of the underlying disease, and which diagnostic criteria would be more appropriate for children. 

ESS has been investigated in critically ill patients and also in adult cancer patients, especially those with lung cancer. Cengiz et al (13) found that 35% of patients had ESS in non-small cell lung cancer. Yasar et al (10) investigated 120 lung cancer patients and ESS was identified in 30 (42 %) of 71 non-small cell lung cancer patients and 22 (44 %) of 49 small-cell lung cancer patients. Tellini et al (26) investigated thyroid hormone levels in 220 cases with malignancy in different organs and found ESS in 58% of the patients. Gao et al (9) reported the incidence of ESS in 188 cases with diffuse large B cell lymphoma as 12.8%. ESS ratios throughout these studies were determined at the time of diagnosis. Knowledge pertaining to ESS in childhood cancer is scarce. To our knowledge, the frequency of ESS in childhood cancers has only been investigated in one study on one type of cancer. Mohn et al (14) investigated ESS prevalence in a small group of seven children with Hodgkin disease and found that five had ESS at the time of diagnosis giving an incidence of 71.4%. ESS was identified in 17.3% of the cases in this study according to criteria 1 and 12% of cases according to criteria 2. Our findings on incidence of ESS was lower than those reported by Tellini et al (26), Cengiz et al (13) and Yasar et al (10) but comparable to results reported by Gao et al (9). While our results indicate a much smaller incidence than was found by Mohn et al (14), this may be due to differences in type of childhood cancer or small group sizes in both our and Mohn’s studies.

Several mechanisms are responsible for ESS. The most important of these is the inhibition of the 5′-deiodinating process in peripheral conversion of T4 to T3 (27,28). ESS is thought to be a result of impaired or decreased peripheral conversion of T4 to T3. However, either an increased conversion of T4 to rT3, and/or a decrease in the ability to degrade rT3 could result in ESS. Since the formation of T3 from T4 and the degradation of rT3 both require 5′-deiodinase, an impairment in the function of this enzyme would result in a decreased ability to form T3 and a reduced ability to further deiodinate rT3 (28). The answer to the questions of what is responsible for low T3 syndrome, whether low T3 syndrome constitutes an adaptive, and thus beneficial response, or whether it aggravates a patient’s condition, is still a matter of debate.

ESS has been reported in a limited number of studies in adult cancer patients and has been considered as an independent predictor of poor prognosis (9,10,13). As the stage of disease increased, ESS was diagnosed more frequently in patients with cancer (9,10,13). In this present study, we saw that thyroid hormone levels did not return to normal value after induction therapy in five patients with ESS who were diagnosed according to fT3 values (criteria 1) and these patients had resistant/relapsed disease. These data show that the event free survival of patients with ESS is worse than non-ESS patients. In addition, our results suggest that patients who did not show a reversion to normal thyroid hormone levels after induction therapy are prone to have relapsed disease. In our opinion patients who are diagnosed as ESS and who do not have normal thyroid hormone levels after induction therapy should be monitored more closely. We cannot comment on the effects of ESS on overall survival because of the short follow-up time in our study.

With only a few exceptions, notably uremia and human immunodeficiency virus (HIV) infection and the closely associated acquired immunodeficiency syndrome (AIDS), serum rT3 concentrations are elevated in ESS (29,30,31). This is thought to be due to a decrease in the conversion of T4 to T3 and rT3 in the catabolic process. We found that the rT3-based ESS was less frequent in our study compared to the group in which ESS was fT3-based. We think that this may be due to the catabolic state of patients diagnosed with cancer. Measurements of TNF levels vary in patients with AIDS and they often have low levels of TNF (32,33). It is thought that this may be related to low rT3 levels in ESS in HIV-positive patients. In our study, we found that TNF-α levels did not correlate with T3 levels in patients with ESS according to criteria 2 (rT3-based ESS). This result may suggest that a low TNF-α level prevents elevation, as in ESS in HIV positive patients. Also, in our study, more relapsing cases were observed in the ESS group based on fT3. These results suggest that fT3-based ESS may be more clinically helpful, as our study and other studies indicate.

Loss of appetite, increased catabolism, feeding disorders and nutritional insufficiencies are thought to be responsible for the development of ESS in cancer patients (9,10,13). Schulte et al (34), in their study on ESS incidence among bone marrow transplant patients, found that ESS cases had lower BMI and serum albumin levels. Tellini et al (26) reported a correlation between ESS and albumin level and degree of weight loss in oncology patients. Cengiz et al (13) showed significant correlations between ESS and nutritional parameters including BMI and serum albumin level. Gao et al (9) showed reduced albumin levels significantly correlated with low T3 syndrome. In contrast to these findings Dişel et al (12) reported that there was no significant difference in weight loss and BMI score between their groups. Reliance on weight measurement can be misleading because of the potential for large tumor mass (>10% body weight) in children with solid tumors (35). This can lead to misinterpretation of weight-based measurements, such as weight for age, weight for height and BMI. In our study, most patients had an intra-abdominal mass, so we could not demonstrate any significant difference in BMI between patients with ESS and those without ESS.

The etiology of ESS is multifactorial. Cytokines, especially pro-inflammatory cytokines such as IL-6, TNF-α and IL-1β, have been suggested as putative mediators of ESS (36,37). It has been shown that these cytokines inhibit the 5’-deiodinase enzyme responsible for the conversion of T4 into T3 in peripheral tissues (36). Investigation of the effects of administering TNF-α and IL-1β to experimental animals and humans confirmed a possible role in the pathogenesis of ESS, with each cytokine inducing critical illness and inducing low serum T3 (36,37,38). Both cytokines also induce IL-6 production. IL-6 is known to exert regulatory effects upon many endocrine systems, either independently, or acting with other cytokines (36,37,38). Acute decreases in T3 and TSH after IL-6 administration have been demonstrated (39). During prolonged administration of IL-6, these effects seemed to be transient. These findings show that IL-6 may, at least in part, mediate the development of ESS, whereas factors other than IL-6 contribute to the persistence of changes in thyroid hormone levels during the chronic phase. TNF-α may also play an important role in pathogenesis of ESS. Administration of recombinant TNF-α to healthy individuals was reported to reproduce thyroid hormone profile resembling ESS (37).

In the present study serum IL-6 and TNF-α levels were higher in patients with ESS. Our results thus support their possible role as endocrine cytokines with a regulatory effect on the thyroid gland. IL-6 and TNF-α levels negatively correlated with fT3 concentration in criteria 1 patients with ESS (fT3 based). However, there was no correlation between TSH and IL-6 and TNF-α. This was not surprising since TSH was maintained within the reference values. Boelen et al (40) measured IL-6 and soluble cytokine receptors for IL-1 and TNF-α in patients with ESS and noted a significant negative correlation between these and circulating T3 levels. Our results corroborate this finding. According to our results, these cytokines may have a role in the onset of ESS in childhood cancer. van der Poll et al (41) showed, even though injection of endotoxin in 18 healthy humans mimicked the thyroid hormone profile of ESS, that the co-infusion of an IL-1 receptor antagonist did not affect the endotoxin-induced changes. This result showed IL-1 may not contribute to the development of ESS. We could not find any correlation between IL-1β and thyroid hormone levels in ESS groups in our study. We therefore suggest that there is no contribution of IL-1β to the development of ESS.

### Study Limitations

There are some limitations of our study that should be noted. The study had a cross-sectional design and had a small sample size. Also, diagnostic heterogeneity makes it difficult to make comparisons between studies and to generalize about the results of our study. Also, we used BMI and serum albumin levels to investigate nutritional status and these have limitations as noted previously. Despite these limitations, to our knowledge this is the first report of ESS in a range of childhood cancers.

## Conclusion

ESS can be present at diagnosis in a range of childhood cancers and thyroid functions should be assessed routinely in pediatric cancer patients at diagnosis. Further studies with larger sample size are needed to confirm the incidence of ESS and the prognostic contribution in childhood cancer.

## Figures and Tables

**Table 1:**
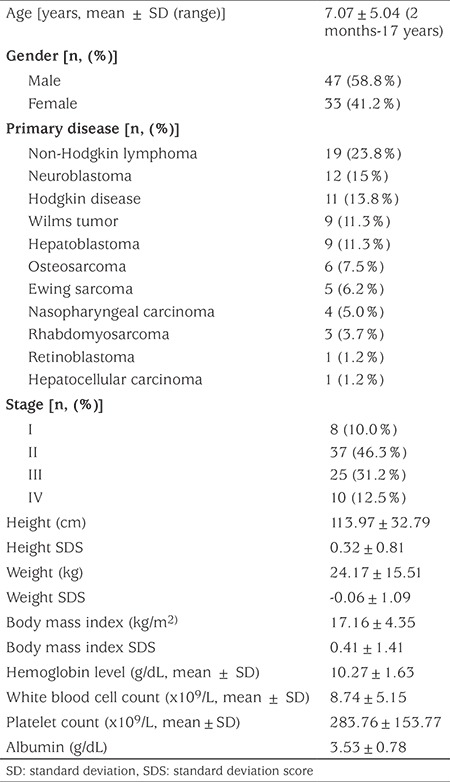
Dermographic and laboratory features of the patients

**Table 2:**
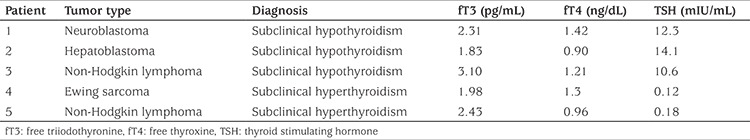
Thyroid function in patients with subclinical hypothyroidism and subclinical hyperthyroidism

**Table 3:**
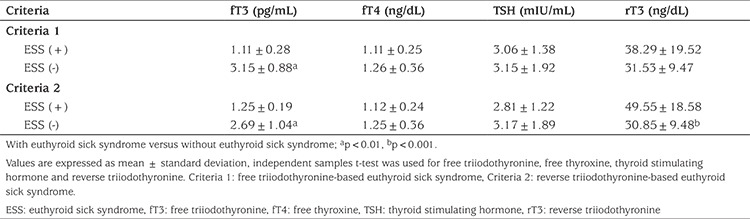
Thyroid function in children according to groups

**Table 4:**
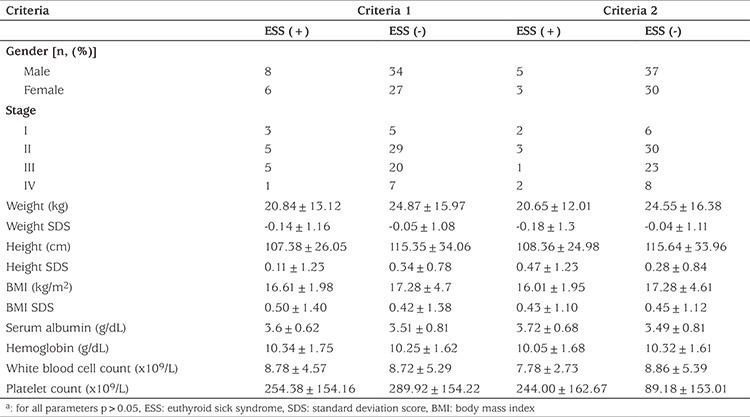
Comparison of demographic and laboratory features of patients by two ESS criteria^a^

**Table 5:**
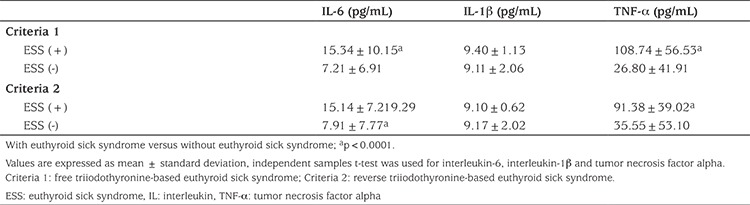
Serum IL-6, IL-1β and TNF-α levels in patients with or without ESS according to groups
